# Supplementary figures and images for: Alzheimer-mutant γ-secretase complexes stall amyloid β-peptide production
Source: eLife. 2025 Feb 11;13:RP102274. doi: 10.7554/eLife.102274 (PMC11813224; doi:10.7554/eLife.102274)

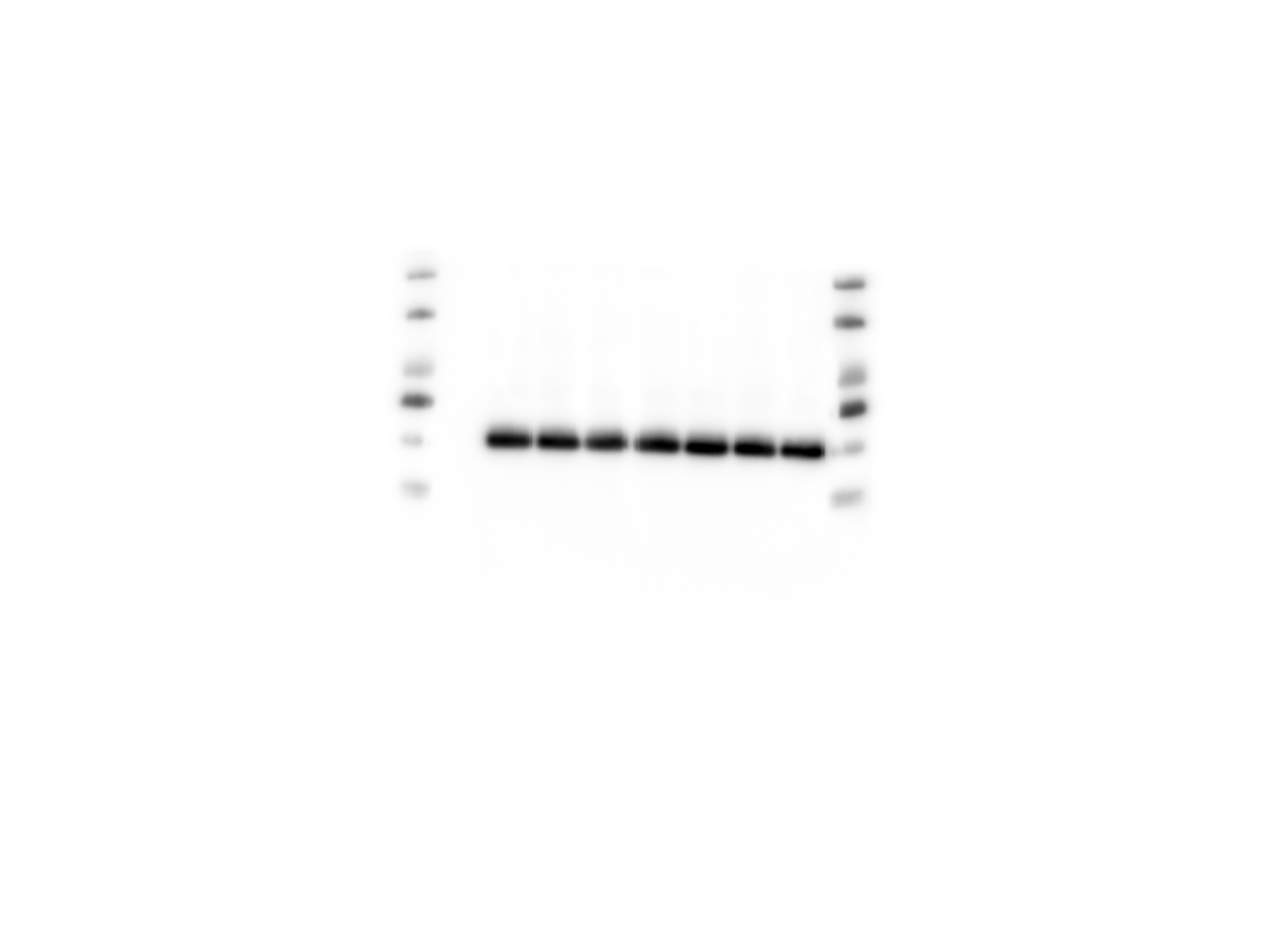

Supplement: Figure 1—figure supplement 2—source data 1. [file elife-102274-fig1-figsupp2-data1.zip › 2023-1230-121904pen2_pub.tif]

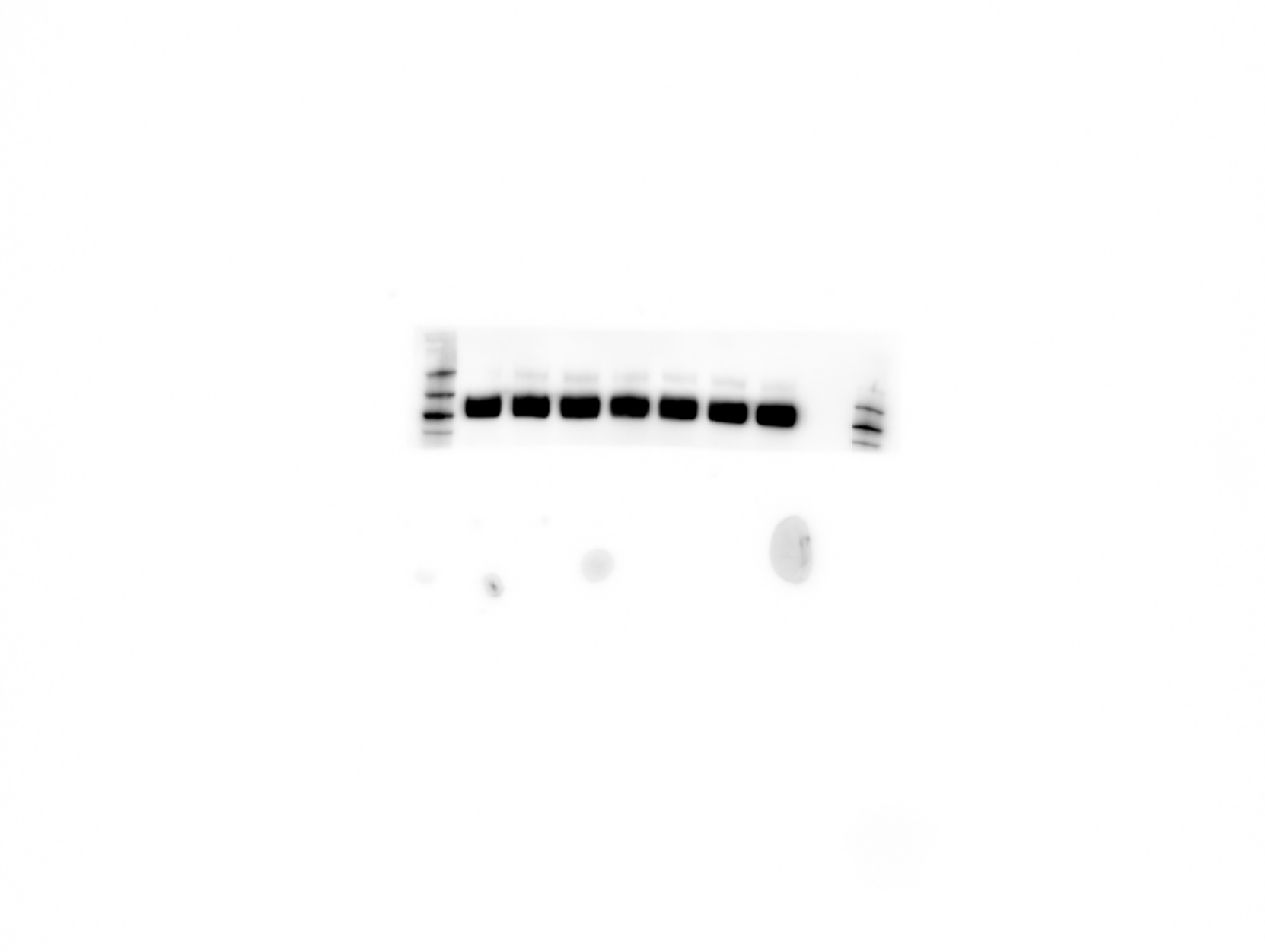

Supplement: Figure 1—figure supplement 2—source data 1. [file elife-102274-fig1-figsupp2-data1.zip › 2023-1230-122411nic_pub.tif]

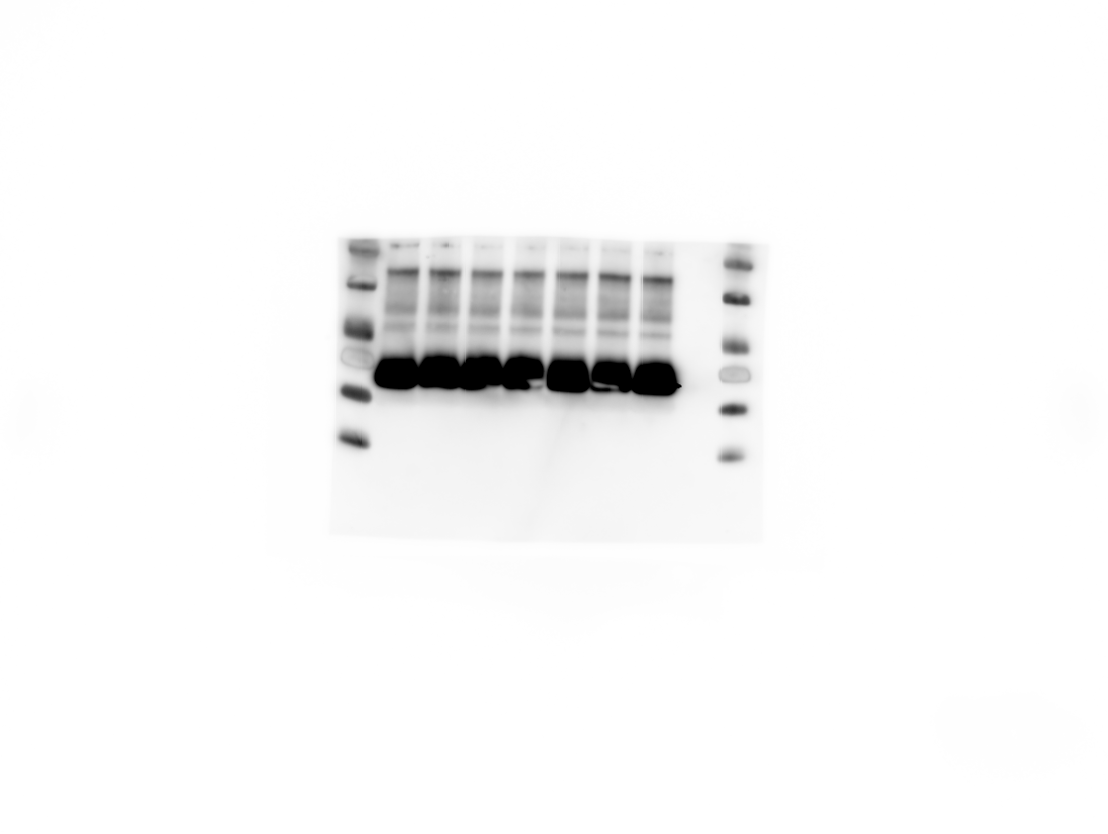

Supplement: Figure 1—figure supplement 2—source data 1. [file elife-102274-fig1-figsupp2-data1.zip › 2023-1231-113334fullps1.tif]

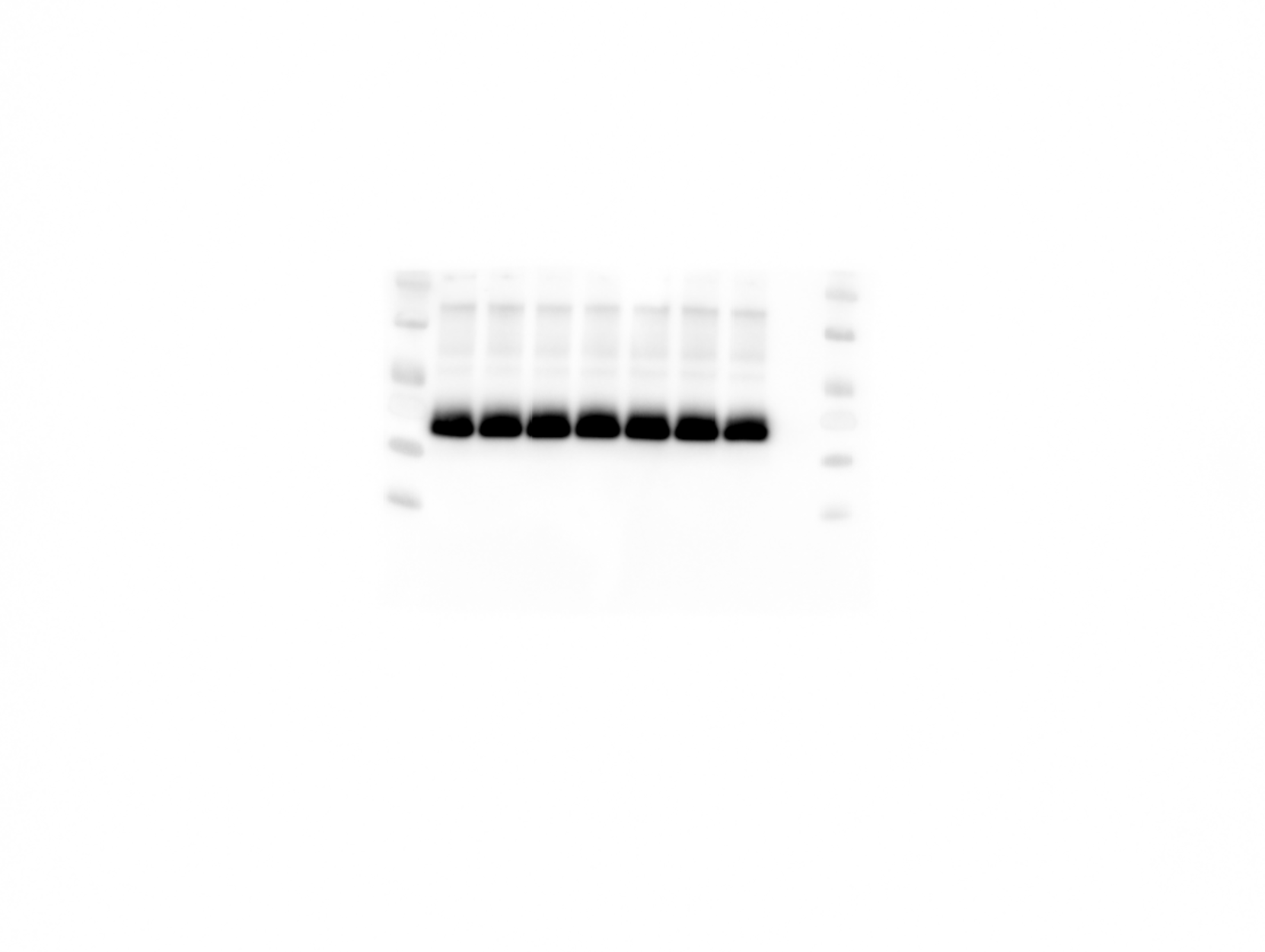

Supplement: Figure 1—figure supplement 2—source data 1. [file elife-102274-fig1-figsupp2-data1.zip › 2023-1231-113442PS1CTF_pub.tif]

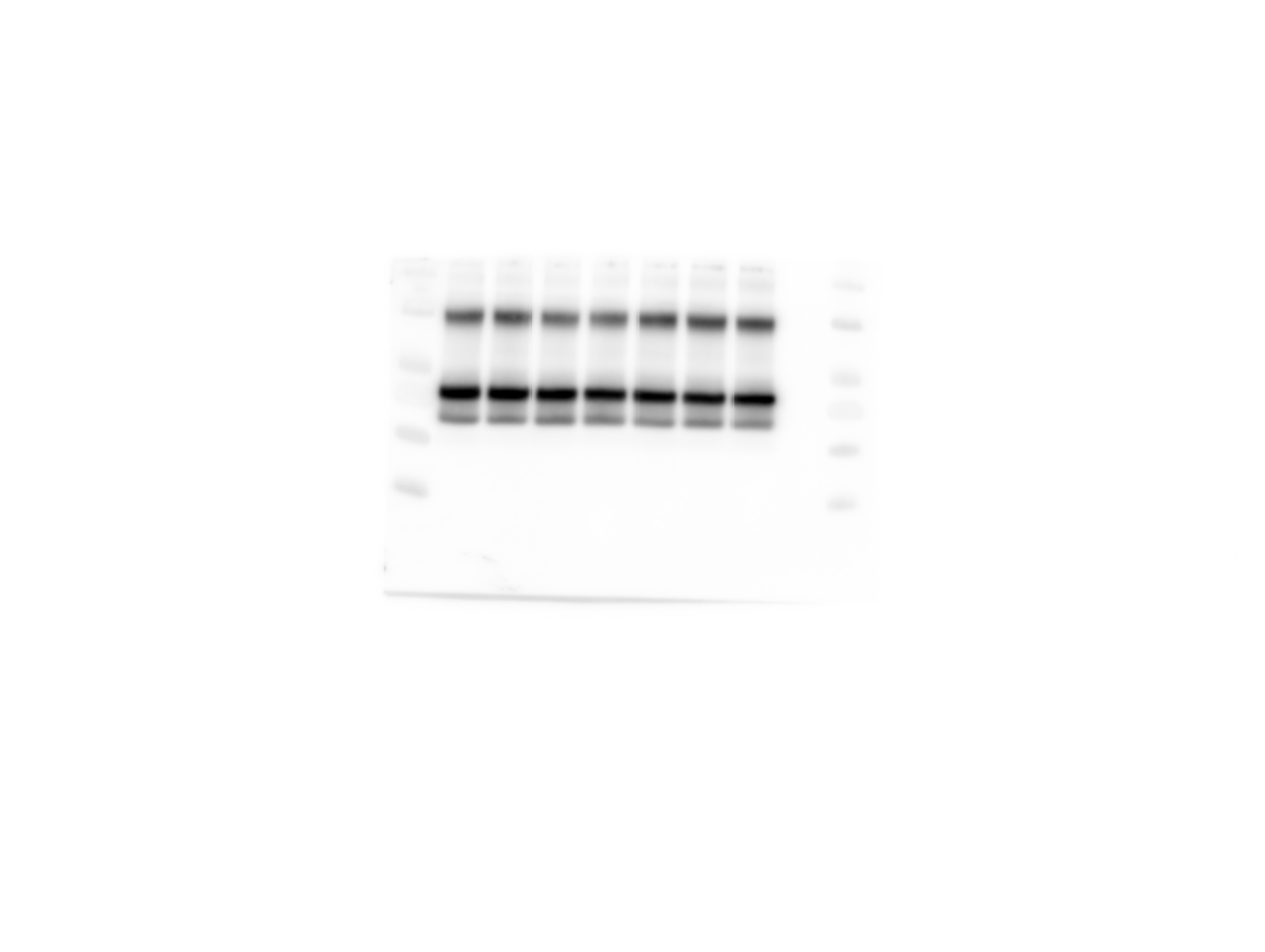

Supplement: Figure 1—figure supplement 2—source data 1. [file elife-102274-fig1-figsupp2-data1.zip › 2024-0104-083729Aph-1_pub.tif]

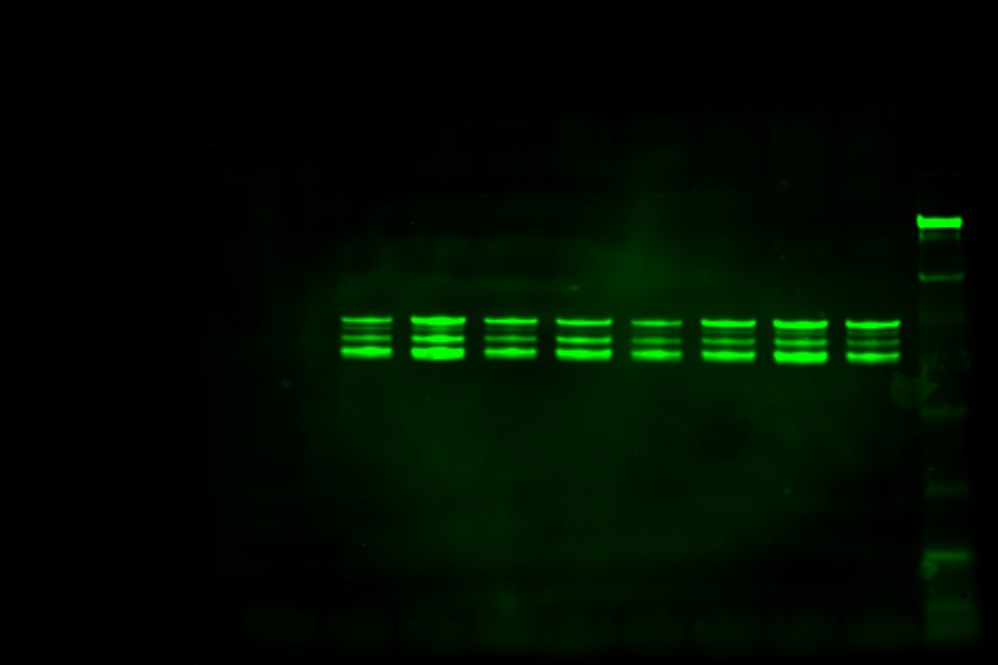

Supplement: Figure 4—figure supplement 1—source data 2. [file elife-102274-fig4-figsupp1-data2.zip › C99 (FLAG).tif]

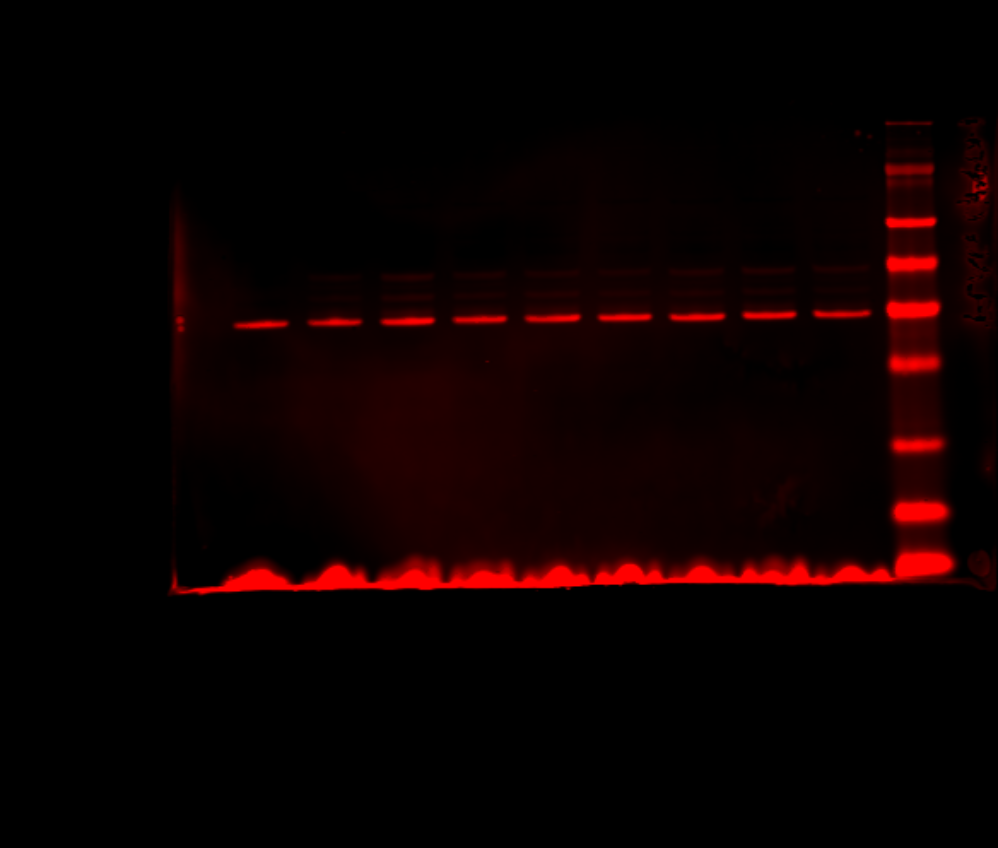

Supplement: Figure 4—figure supplement 1—source data 2. [file elife-102274-fig4-figsupp1-data2.zip › GAPDH.tif]

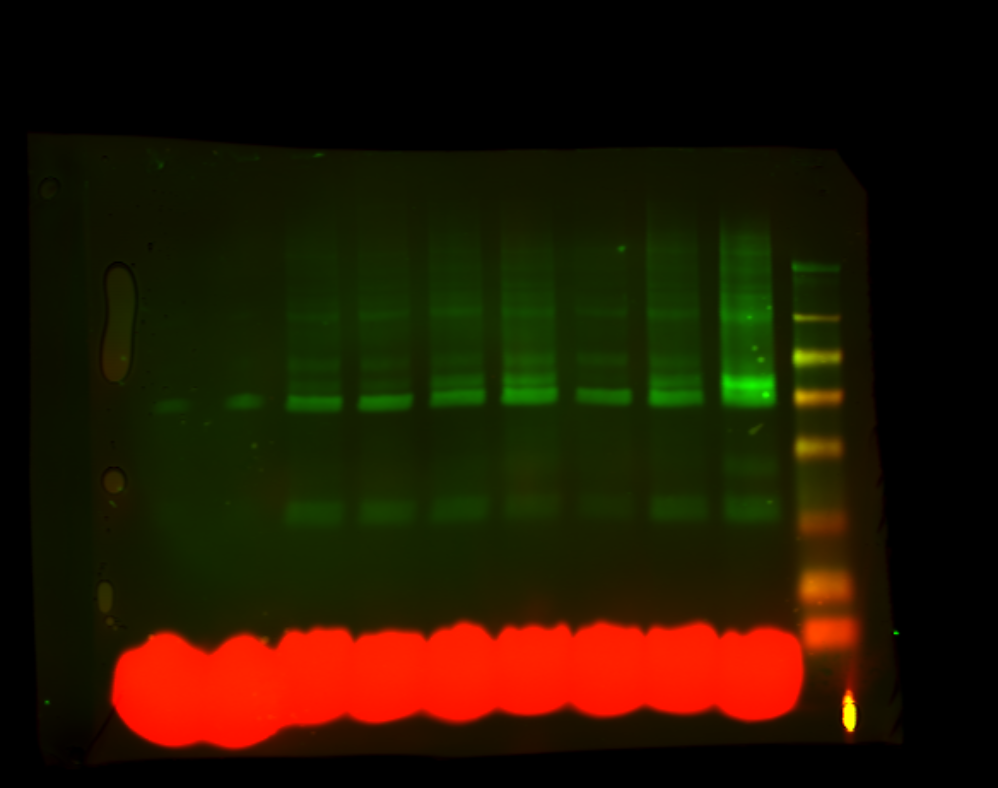

Supplement: Figure 4—figure supplement 1—source data 2. [file elife-102274-fig4-figsupp1-data2.zip › PSEN1.tif]
