# Supplementary material for: Alzheimer-mutant γ-secretase complexes stall amyloid β-peptide production
Source: eLife. 2025 Feb 11;13:RP102274. doi: 10.7554/eLife.102274 (PMC11813224; doi:10.7554/eLife.102274)
Supplement: Figure 1—figure supplement 2—source data 2. [file elife-102274-fig1-figsupp2-data2.zip › Figure 1-Figure supplement 2-source data 2.pptx]

## Slide 1
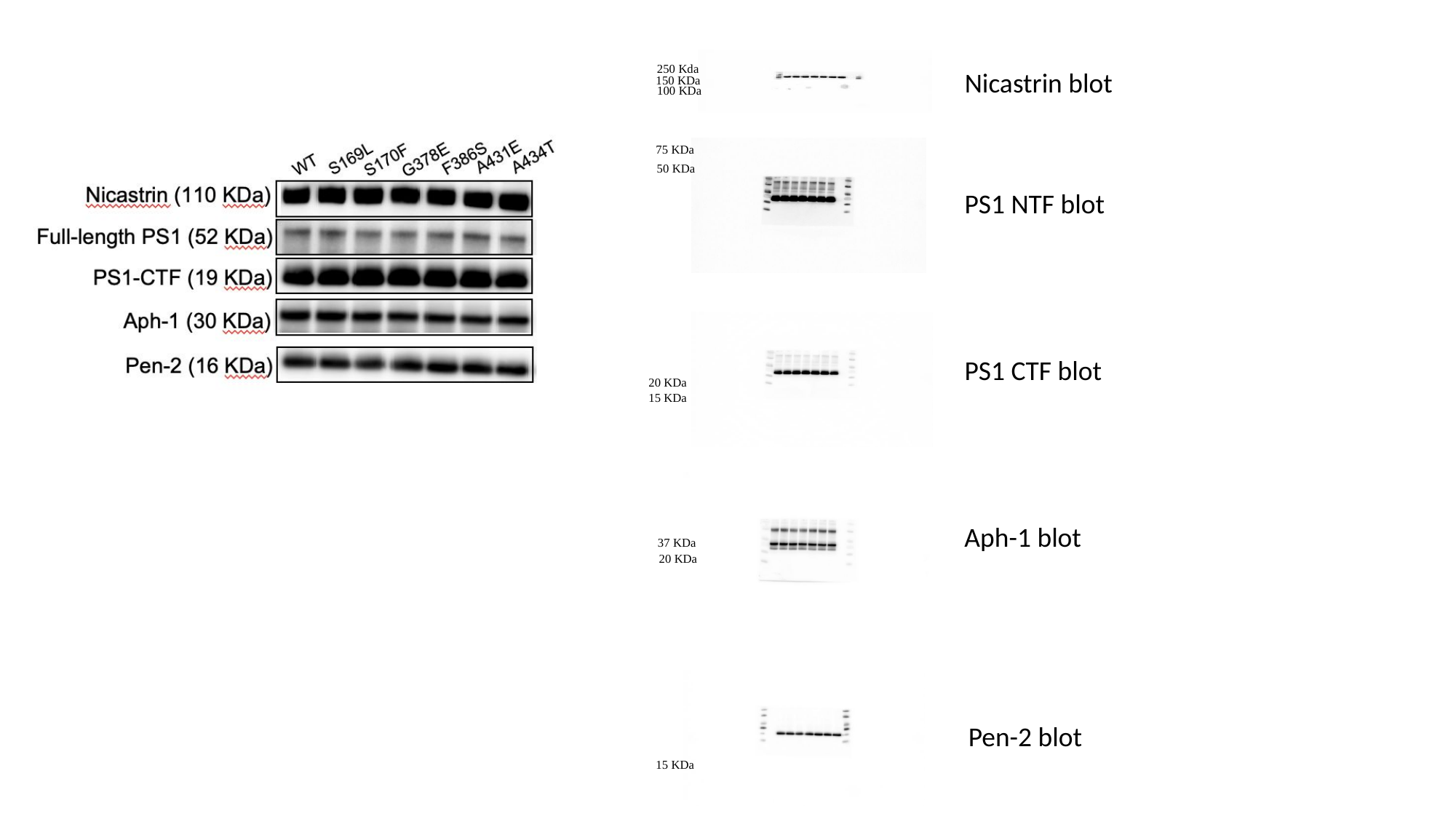

250 Kda
Nicastrin blot
150 KDa
100 KDa
75 KDa
50 KDa
A434T
A431E
S169L
F386S
PS1 NTF blot
S170F
G378E
WT
Nicastrin (110 KDa)
Full-length PS1 (52 KDa)
PS1-CTF (19 KDa)
Aph-1 (30 KDa)
Pen-2 (16 KDa)
PS1 CTF blot
20 KDa
15 KDa
Aph-1 blot
37 KDa
20 KDa
Pen-2 blot
15 KDa
