# Supplementary material for: Alzheimer-mutant γ-secretase complexes stall amyloid β-peptide production
Source: eLife. 2025 Feb 11;13:RP102274. doi: 10.7554/eLife.102274 (PMC11813224; doi:10.7554/eLife.102274)
Supplement: Figure 4—figure supplement 1—source data 3. [file elife-102274-fig4-figsupp1-data3.zip › Arafi et al., eLife Figure 4-supplemental 1.pptx]

## Slide 1
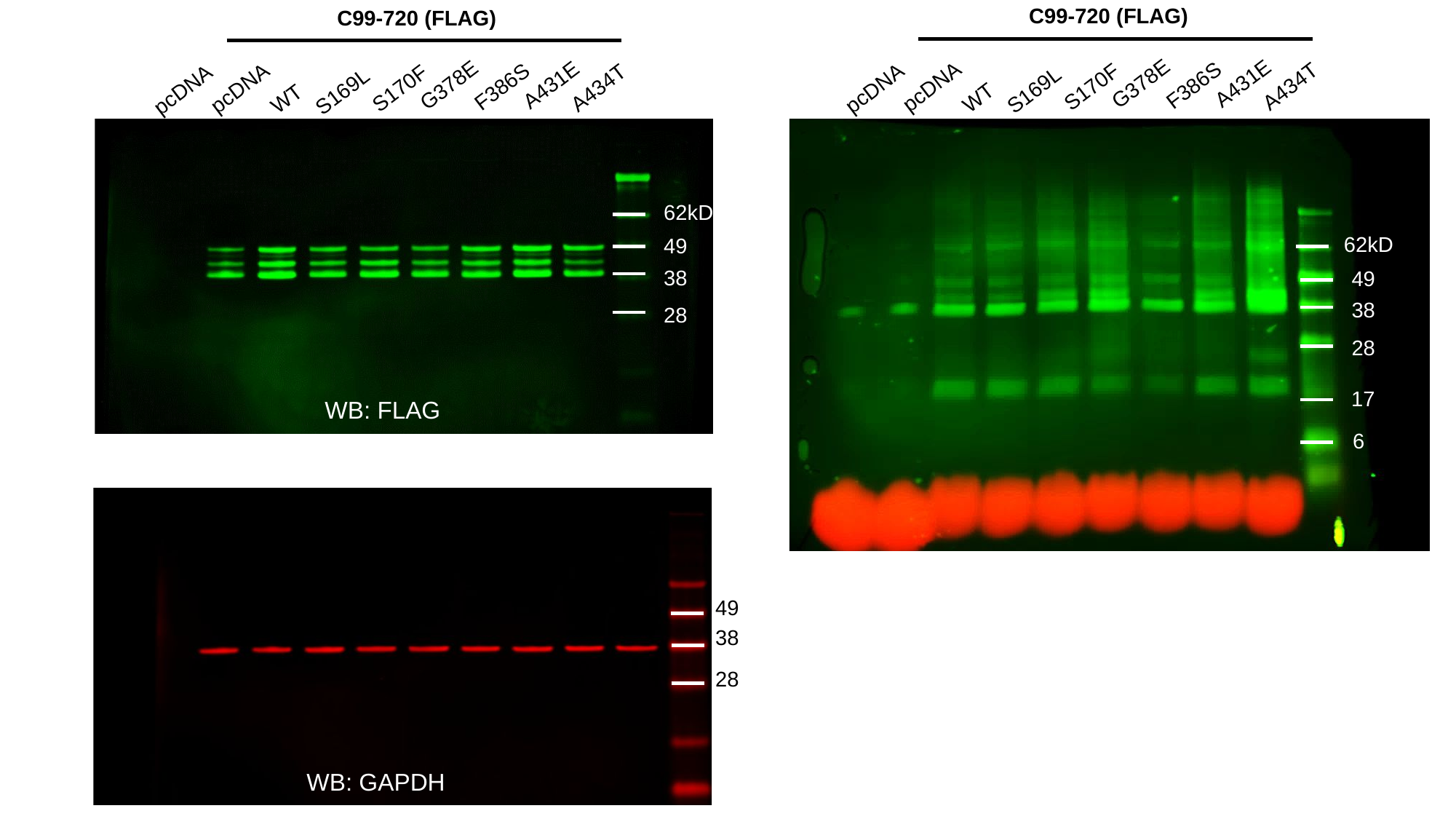

C99-720 (FLAG)
C99-720 (FLAG)
A431E
G378E
A431E
F386S
G378E
A434T
F386S
S170F
A434T
S170F
S169L
S169L
pcDNA
pcDNA
pcDNA
pcDNA
WT
WT
62kD
62kD
49
38
49
38
28
28
17
WB: FLAG
6
49
38
28
WB: GAPDH
