# Supplementary material for: Alzheimer-mutant γ-secretase complexes stall amyloid β-peptide production
Source: eLife. 2025 Feb 11;13:RP102274. doi: 10.7554/eLife.102274 (PMC11813224; doi:10.7554/eLife.102274)
Supplement: Supplementary file 1. — Cross-reactivity of Aβ43 with Aβ42 in ELISAs. Various concentrations of Aβ43 (ranging from 15.63 pg/mL to 1,000,000 pg/mL) were tested using Aβ42-specific ELISA kits. The instrument readings for each concentration are displayed, indicating significant cross-reactivity starting at 250 pg/mL (0.06 nM) of Aβ43. (Note: ‘OF’ stands for overflow.) [file elife-102274-supp1.docx]

| **Concentration of**  **Aβ43 (pg/mL)** | **Cross reactivity**  **(Read: pg/mL)** |
| --- | --- |
| **15.63** | 8.9 |
| **31.25** | 10.5 |
| **62.5** | 12.0 |
| **125** | 14.2 |
| **250** | 28.8 |
| **500** | 67.4 |
| **1000** | 203.3 |
| **2500** | 867.4 |
| **5000** | OF |
| **10000** | OF |
| **20000** | OF |
| **200000** | OF |
| **1000000** | OF |
