# Supplementary material for: Alzheimer-mutant γ-secretase complexes stall amyloid β-peptide production
Source: eLife. 2025 Feb 11;13:RP102274. doi: 10.7554/eLife.102274 (PMC11813224; doi:10.7554/eLife.102274)
Supplement: Supplementary file 2. — Cross-reactivity of Aβ43 with Aβ40 in ELISAs. Different concentrations of Aβ43 (ranging from 7.8 to 1,000,000 pg/mL) were assessed using ELISA kits specific for Aβ40. The resulting instrument readings for each concentration are presented, revealing cross-reactivity beginning at 500 pg/mL (0.12 nM) of Aβ43. [file elife-102274-supp2.docx]

| **Concentration of**  **Aβ43 (pg/mL)** | **Cross reactivity**  **(Read: pg/mL)** |
| --- | --- |
| **7.8** | 15.8 |
| **15.63** | 11.4 |
| **31.25** | 22.6 |
| **62.5** | 7.1 |
| **125** | 15.0 |
| **250** | 20.7 |
| **500** | 32.5 |
| **1000** | 41.6 |
| **50000** | 93.4 |
| **100000** | 104.2 |
| **200000** | 116.3 |
| **1000000** | 117.4 |
